# Dinuclear Metal-Mediated Homo Base Pairs with Metallophilic Interactions: Theoretical Studies of G_2_M_2_^2+^ (M = Cu, Ag, and Au) Ions

**DOI:** 10.1038/s41598-017-14259-2

**Published:** 2017-11-02

**Authors:** Guo-Jin Cao

**Affiliations:** Institute of Molecular Science, Shanxi University, Taiyuan, 030006 China

## Abstract

Dinuclear metal-mediated homo base pairs are interesting clusters with highly symmetric structures and significant stabilities. The geometric and electronic structures of G_2_M_2_^2+^ (G = Guanine, M = Cu, Ag or Au) cluster ions were studied with quantum chemical calculations. The lowest-energy isomers of G_2_M_2_^2+^ cluster ions have C_2h_ symmetries with an approximately antiparallel alignment of two sets of N-M∙∙∙O groups being formed in the planar structures. The M-M distances are shorter than the sum of van der Waals radii of corresponding two homo coinage metal atoms, showing that metallophilic interactions significantly exist in these complexes. They have the large HOMO−LUMO gaps of about 5.80 eV at the DFT level and the bond dissociation energies of more than 5.60 eV at the DFT/B3LYP level, indicating that these cluster dications are highly stable. The second lowest-energy isomers stabilized by an approximately parallel alignment of one set of O-M-O group and one set of N-M-N group are found to be close to the lowest-energy isomers in energy. The barrier between the two isomers of G_2_M_2_^2+^ cluster ions is significantly large, also showing that these lowest-energy isomers are very stable.

## Introduction

Metal ion-base pair complexes have attracted tremendous attention because of their importance in the development of nanotechnology and biotechnology^1–8^. They have significantly thermal duplex stabilization as well as the conductive property. It has been reported that metal-mediated base pairs can be used as nanowires^2^, novel sensors competent to detect metal ions in aqueous solutions^9^, bifacial nucleobases^10^, and logic gates^11^. In these metal ion-base pair complexes, dual complementary hydrogen bonds between nucleobases are replaced by the metal-nucleobase bonds^12^. Dinuclear metal ion-mismatched base pairs have become an interesting class of metal ion-base pair complexes in recent years. There have been many theoretical and experimental investigations on the complexes of dinuclear silver ions and mismatched base pairs^13–19^. More recently, dinuclear Cu^II^ and Hg^II^ complexes containing an artificial nucleobase (9-ethyl-1, N6-ethenoadenine) have been prepared and their crystal structures have been determined by Mandal *et al*.^20–22^. Their studies show that dinuclear *T-Hg* ^*II*^_2_ *-εA* base pair has two sets of parallel M-N bonds with anionic ligands partially compensating the charge of metal ions.

The terms “aurophilic bonding” and “aurophilic interactions” for Au^+^-Au^+^ interactions were introduced by Schmidbaur *et al*. in 1988^23,24^. The weaker attractive interactions between coinage metal cations with closed-shell configurations in coinage metal compounds have been termed as “numismophilicity” (from the Latin word “numisma” meaning a coin) by Vicente *et al*.^25^ in 1993, and then extended as “metallophilic interactions” for closed-shell interactions of metal atoms by Pyykkö^26^ in 1994. Metallophilic interactions are stronger than van der Waals forces but weaker than covalent bonding, close to hydrogen bonding in energy, influencing significantly a number of structural and characteristics of metal compounds. Concerning the intensities of metallophilic interactions, it is generally believed that aurophilic interactions should be stronger than argentophilic bonding due to the strong relativistic effects of Au. Ag ≈ Au seems more reasonable because Ag-Ag distances are very close to Au-Au distances in a family of analogous complexes^27,28^.

In our preceding papers, we have investigated the coinage metal-nucleobase complexes by photoelectron spectroscopy, photodissociation, and theoretical calculations^29–31^. G_2_M^+^ (G = guanine, M = Cu, Ag or Au) cluster ions prefer planar structures with the metal cations interacting with the N7 atoms of guanine. In my previous papers, metal-to-ligand (MLCT), ligand-to-metal (LMCT), intraligand (LL) charge transfer processes have been found to exist in the photodissociation of G_2_M^+^ ions. The bonding and electronic structures of dinuclear coinage metal-mediated base pairs containing divalent metal ions remain elusive. Cu^+^, Ag^+^, and Au^+^ cations have a ^1^S electronic ground state with the valence electron configuration nd^10^(n + 1)s^0^(n + 1)p^0^. This poses the question about the electron configuration of metal cations in G_2_M_2_^2+^ complexes and the nature of chemical bonding in the metallophilic and nucleobase-M^+^-nucleobase interactions. Gold exhibits larger relativistic effects than those of Cu and Ag, such as 5d relativistic expansion and destabilization, and 6s relativistic stabilization and contraction^32–39^. It is interesting to pinpoint whether dinuclear coinage metal-mediated homo base pairs are also stable and in which direction gold-mediated base pairs might deviate from the copper or silver analogues. In this work, we investigate the whole G_2_M_2_^2+^ series for M = Cu, Ag and Au and examine the chemical bonding using various theoretical methods. The calculated results for the G_2_M_2_^2+^ complexes provide new aspects for chemical bonding of the coinage metals and help to find potential applications of metal-mediated base pairs complexes.

## Theoretical results

**Figure 1:**
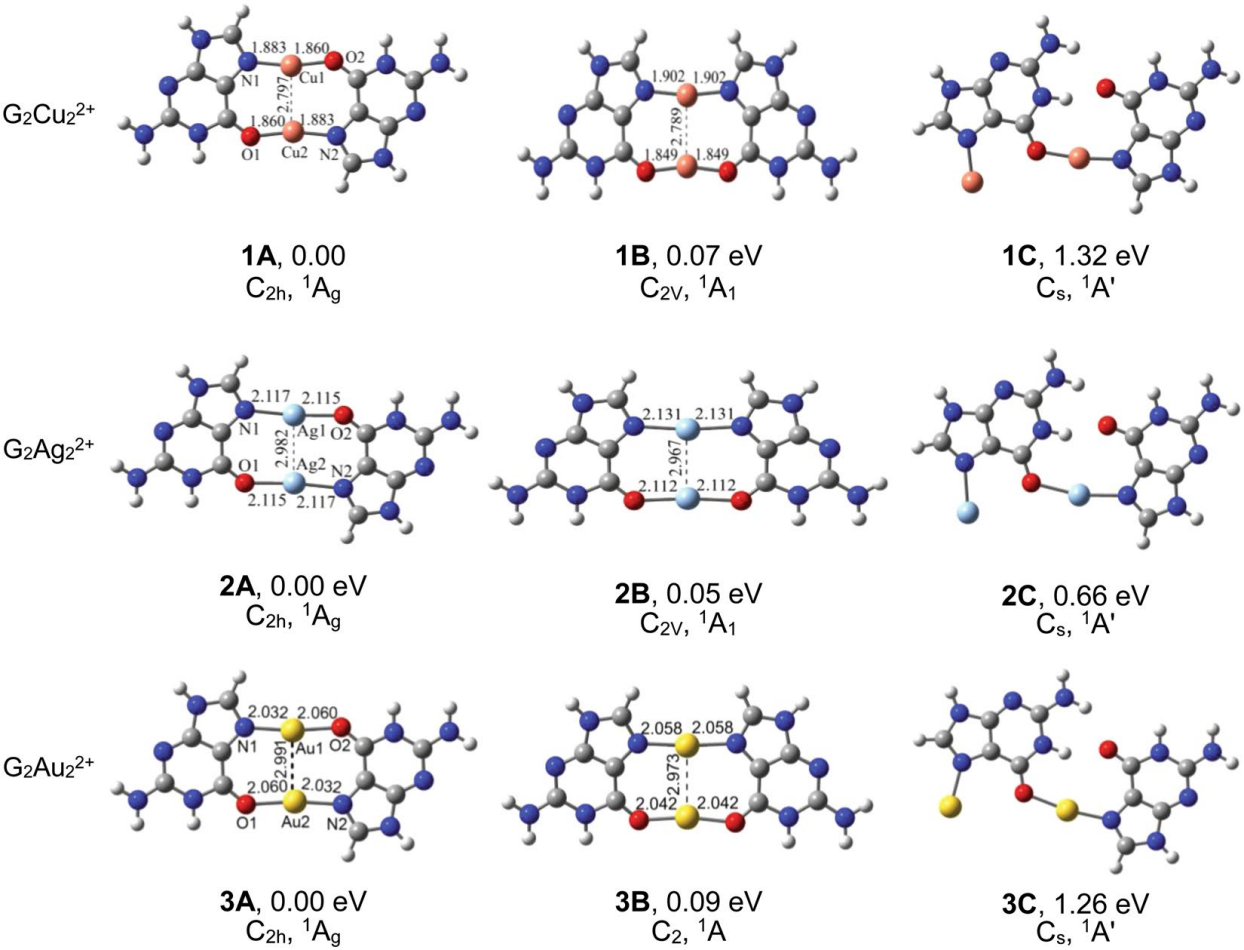
Structures and relative energies of the low-lying isomers of G_2_M_2_^2+^ cluster ions. The bond distances are in angstroms.

In search of the global minimum of G_2_M_2_^2+^ (M = Cu, Ag or Au) cluster dications, various possible initial geometric structures have been considered. The canonical (K-N9H) tautomer^40^ of guanine was taken into account in the search of their low-energy isomers. The structures, bond lengths, and N-M-O angles of low-energy isomers of these complexes are displayed in Fig. 1 and Table 1. Relative energies (eV), symmetries and electronic states of the low-energy isomers of G_2_M_2_^2+^ cluster ions are shown in Table 2. The optimized structures of G_2_M_2_^+^ cluster ions are in singlet spin states. The triplet and quintet spin states are much less stable than the singlet spin states in energy. All of these complexes are planar structures in guanine-M_2_-guanine style, containing one set of nearly linear N–M–N and one group of roughly linear N–M–O bonds.

**Table 1:** Optimized Bond Lengths and ∠N-M-O Angles of the Lowest-lying Isomers of G_2_M_2_^2+^ Cluster Ions at the DFT Level^a^.

	Distance	Vacuum	COSMO
G_2_Cu_2_^2+^	Cu-Cu	2.797	2.680
	Cu-N	1.883	1.852
	Cu-O	1.860	1.844
	∠N-Cu-O	168.7	165.3
G_2_Ag_2_^2+^	Ag-Ag	2.982	2.815
	Ag-N	2.117	2.093
	Ag-O	2.115	2.102
	∠N-Ag-O	174.1	169.7
G_2_Au_2_^2+^	Au-Au	2.991	2.906
	Au-N	2.032	2.009
	Au-O	2.060	2.040
	N-Au-O	173.0	170.6

^a^The bond distances are in angstroms and the bond angles are in degrees.

**Table 2:** Relative Energies (eV), Symmetries and Electronic States of the Low-energy Isomers of G_2_M_2_^2+^ Cluster Ions.

	Isomers	Sym.	State	ΔE (eV)		
				PBE/TZ2P	CAMY-B3LYP	COSMO
G_2_Cu_2_^2+^	1A	C_2h_	^1^A_g_	0.00	0.00	0.00
	1B	C_2V_	^1^A_1_	0.08	0.07	0.04
	1C	C_s_	^1^A′	1.34	1.32	1.28
G_2_Ag_2_^2+^	2A	C_2h_	^1^A_g_	0.00	0.00	0.00
	2B	C_2V_	^1^A_1_	0.05	0.05	0.02
	2C	C_s_	^1^A′	0.68	0.66	0.62
G_2_Au_2_^2+^	3A	C_2h_	^1^A_g_	0.00	0.00	0.00
	3B	C_2_	^1^A	0.10	0.09	0.06
	3C	C_s_	^1^A′	1.28	1.26	1.23

As seen in Fig. 1, all the lowest-energy isomers **1A**, **2A** and **3A** of G_2_M_2_^2+^ cations have perfectly planar structures with C_2h_ symmetries. An approximately antiparallel orientation of two sets of N-M∙∙∙O groups are formed in these isomers **1A**, **2A** and **3A**. The N-M∙∙∙O units have nearly linear arrangements and the order of their angles is Cu < Au < Ag (∠N-Cu-O = 168.7°, ∠N-Ag-O = 174.1°, ∠N-Au∙∙∙O = 173.1°). The Cu-N distances are near 1.893 ± 0.010 Å, and Cu-O distances are 1.855 ± 0.006 Å, depending on the structures. The order of M-N or M-O distances is Cu < Au < Ag, in agreement with the order of the atomic radii increments suggested by Pyykkö^41^ (Cu ~ 1.12 Å, Ag ~ 1.28 Å, Au ~ 1.24 Å). Considering the Au-Au distances, aurophilic Au-Au distances can vary from 2.85 Å to 3.50 Å depending on their strength^28^. The Au-Au distance is 2.991 Å in the lowest-energy isomer **3A**, much larger than the single-bond covalent radii (2.48 Å) recommended by Pyykkö^41^, indicating that there is hardly strong Au–Au bonding interaction except the aurophilic interaction. The Ag-Ag distance is near 2.982 Å in isomer **2A**, significantly longer than the distance of a Ag-Ag single bond (2.53 Å), but shorter than the sum of van der Waals radii (3.44 Å)^27^. It is very close to the Au-Au distance in isomer **3A**, showing that the metallophilic bonding for Ag and Au is very similar. The Cu-Cu distances in isomer **1A** are near 2.797 Å, close to the distances of the dimer [{H_3_PAgCl}_2_] with cuprophilic interactions (2.637–2.809 Å at DFT and ab initio levels)^42^. Concerning metal-metal vibrations, the Cu-Cu vibrational harmonic frequency of isomer **1A** is 122.5 cm^−1^ (force constant F = 0.33 mdyn•Å^−1^). The Ag-Ag vibrational frequency of isomer **2A** is 101.1 cm^−1^ (force constant F = 0.47 mdyn•Å^−1^), in accordance with the Ag-Ag vibrations (75–125 cm^−1^) of crystals of Tl[Ag(CN)_2_] detected by a detailed Raman experiment^43^. The Au-Au vibrational frequency of isomer 3 A is 94.8 cm^−1^ (force constant F = 0.57 mdyn•Å^−1^). The order of M-M stretching frequencies is Cu > Ag > Au.

The second lowest-energy isomers **1B**, **2B**, and **3B** of G_2_M_2_^2+^ cluster ions can be considered as derived from isomers **1A**, **2A**, and **3A**, respectively, by revolving guanine about 180° round the C_2_ axis. They are stabilized by an approximately parallel orientation of one set of O-M-O group and one set of N-M-N group, close to the lowest-lying isomers in energy at the DFT level. One note that isomer **3B** has a nonplanar structure with C_2_ symmetry. The M-M distances in isomers **1B-3B** are slightly shorter than those in isomer **1A-3A**. The third lowest-energy isomers of G_2_M_2_^2+^ cluster ions have planar structures with one metal ion involving in the formation of one set of O-M-N group and another metal ion binding to N7 atom of guanine. Isomers **1C**, **2C**, and **3C** are much higher in energy than the lowest-lying isomers **1A**, **2A**, and **3A**. When the aqueous solvent environment is roughly modeled using the COSMO solvent model, the energy differences of two lowest-energy isomers become closer relative to those in the vacuum environment. All the bond lengths become slightly shorter and the order of ∠N-M-O angles becomes Cu < Ag < Au.

Effective atomic charges of the lowest-energy isomers (**1A**, **2A**, and **3A**) of G_2_M_2_^2+^ (M = Cu, Ag, and Au) cluster ions from various population schemes are displayed in Table 3. The charges from the multipole-deformation prescription are slightly larger than those from the Hirshfeld or Voronoi Deformation Density (VDD) prescriptions. Our studies have showed that the Voronoi and Hirshfeld density partitionings are consistent with the chemical shifts of the core-level energies of atoms^44^. According to the Hirshfeld or VDD prescriptions, the coinage metals Cu, Ag, and Au carry large positive charges between 0.18 and 0.34, while the nitrogen and oxygen atoms bonded to the metal atoms carry negatively charges. Thus, strong electrostatic attraction exist in the interactions between the metal atoms and N or O atoms, while the electron-acceptor strength of coinage metal atoms has the order Au > Cu > Ag.

**Table 3:** Effective Atomic Charges of the Lowest-energy Isomer (1 A, 2 A, and 3 A) of G_2_M_2_^2+^ Cluster Ions.

	M	Hirshfeld charge	Voronoi charge	MDC-q
G_2_Cu_2_^2+^	Cu1, Cu2	0.29	0.27	0.39
	N1, N2	−0.11	−0.13	−0.40
	O1, O2	−0.21	−0.22	−0.42
G_2_Ag_2_^2+^	Ag1, Ag2	0.34	0.30	0.53
	N1, N2	−0.11	−0.13	−0.50
	O1, O2	−0.23	−0.23	−0.49
G_2_Au_2_^2+^	Au1, Au2	0.22	0.18	0.43
	N1, N2	−0.08	−0.09	−0.50
	O1, O2	−0.20	−0.19	−0.48

To understand the relative stabilities of the lowest-lying isomers and the second lowest-energy isomers of G_2_M_2_^2+^ cluster ions, the possible transition barriers were calculated between them using the Berny method at the DFT/B3LYP level. Obtained transition-state structures were confirmed to connect the correct reactants and products by intrinstic reaction coordinate (IRC). Vibrational analyses show that there is only one imaginary frequency in the transition-state structures. The lowest transition states for the isomers of G_2_M_2_^2+^ cluster ions were determined with conversion barriers of more than 1.00 eV. The barriers between the two low-lying isomers of G_2_M_2_^2+^ cluster ions are significantly large, and it is hard for them to convert at room temperature.

## Discussion

The Cu 3d-derived valence MOs and lowest unoccupied molecular orbital (LUMO) 4a_u_ of the lowest-energy isomer of G_2_Cu_2_^2+^ cation are displayed in Figure S1 and Table 4. The LUMO 4a_u_ has 95% contribution from the 2p characters of C, N and O atoms, while the 4p orbital of Cu just has 5% contribution to the LUMO. The 3d orbitals of Cu atoms have 90% contribution to the HOMO 5b_u_ (−11.2 eV), while the 2p orbitals of N and O atoms just have 10% contribution to the HOMO. Three sets of energetically degenerate 4a_g_, 2b_g_, 4b_u_ MOs, and one set of 2a_u_ MO are mainly of the 3d orbitals of Cu atoms. The ds hybridizations of the Cu atoms are mainly involved in three sets of 2a_g_, 3a_g_, and 3b_u_ MOs. Seven sets of 3b_g_, 3a_u_, 1b_g_, 2b_u_, 1a_u_, 1a_g_, and 1b_u_ MOs are the combinations of the 3d orbitals of two Cu atoms and the 2p orbitals of C, N, and O atoms. From these analyses, the cuprophilic interaction occurs mainly through the mutual stabilization of nine sets of 3a_u_, 4a_g_, 2b_g_, 4b_u_, 3a_g_, 3b_u_, 2a_u_, 2a_g_, and the highest occupied 5b_u_ MOs.

**Table 4:** Characters, Orbital Energies (in eV), and AO Contributions in % (Largest is Bold) of the Cu 3d-derived MOs and Lowest Unoccupied MO of the Lowest-lying Isomer of G_2_Cu_2_^2+^ dication.

MO	type	ε	occ	Cu(d)	Cu(s)	Cu(p)	N(p)	O(p)	C(p)
1b_u_	Cu(dsp) + C(2p) + N(2p) + O(2p)	−16.3	2	16	2	**3**	**63**	3	13
1a_g_	Cu(dsp) + C(2p) + N(2p) + O(2p)	−16.1	2	10	3	**2**	**63**	7	15
1a_u_	Cu(d) + C(2p) + N(2p) + O(2p)	−14.6	2	11	—	—	**63**	8	18
2b_u_	Cu(d) + C(2p) + N(2p) + O(2p)	−14.1	2	20	—	—	8	**66**	6
2a_g_	Cu(dsp)	−12.6	2	**85**	13	2	—	—	—
1b_g_	Cu(d) + N(2p) + O(2p)	−12.2	2	**52**	—	—	31	15	2
2a_u_	Cu(d)	−12.2	2	**100**	—	—	—	—	—
3b_u_	Cu(ds)	−12.1	2	**95**	5	—	—	—	—
3a_g_	Cu(ds)	−11.9	2	**94**	6	—	—	—	—
4b_u_	Cu(d)	−11.9	2	**100**	—	—	—	—	—
2b_g_	Cu(d)	−11.9	2	**100**	—	—	—	—	—
4a_g_	Cu(d)	−11.8	2	**100**	—	—	—	—	—
3a_u_	Cu(d) + C(2p) + N(2p) + O(2p)	−11.8	2	**83**	—	—	10	4	3
3b_g_	Cu(d) + C(2p) + N(2p) + O(2p)	−11.3	2	31	—	—	**33**	9	27
5b_u_	Cu(d) + N(2p) + O(2p)	−11.2	2	**90**	—	—	3	7	—
4a_u_	Cu(p) + C(2p) + N(2p) + O(2p)	−5.6	0	—	—	5	27	9	**59**

The LUMO 6a_u_ (−5.4 eV) of G_2_Ag_2_^2+^ cluster ions contains 95% C, N, and O 2p, and 5% Ag 5p characters (Table 5 and Figure S2), similar to the lowest unoccupied 4a_u_ MO of Ag’s lighter homologue G_2_Cu_2_^2+^. The 2p orbitals of N and O atoms have 97% contribution to the HOMO 5b_g_ (−11.4 eV), while the 3d orbitals of Ag atoms just have 3% contribution to the HOMO. The characters and bonding of the highest occupied 5b_g_ MO of G_2_Ag_2_^2+^ dication are completely different from those of the highest occupied 5b_u_ MO of Ag’s lighter homologue. Seven sets of 6a_g_, 4b_g_, 5a_u_, 5a_g_, 1b_g_, 1a_u_, and 1a_g_ MOs have major contribution from N 2p character and minor contribution from the 4d orbitals of Ag atoms, 2p orbitals of C and O atoms, while six sets of 1b_u_, 3a_g_, 2b_g_, 4a_g_, 3b_u_, and 4b_u_ MOs have significant contribution from the 4d orbitals of Ag atoms and only little contribution from 2p orbitals of C, N and O atoms. The argentophilic interaction occurs mainly through the mutual stabilization of three sets of low 1b_u_, 2a_g_ and 2a_u_ MOs, two sets of nearly degenerate 3b_g_ and 2b_u_ MOs, and two sets of 4a_g_, and 4b_u_ MOs, and also a little stabilized by two sets of 5a_g_ and 6a_g_ MOs.

**Table 5:** Characters, Orbital Energies (in eV), and AO Contributions in % (Largest is Bold) of the Ag 4d-derived MOs and Lowest Unoccupied MO of the Lowest-lying Isomer of G_2_Ag_2_^2+^ Cluster ion.

MO	ε	occ	Ag(d)	Ag(s)	Ag(p)	N(p)	O(p)	C(p)
1a_g_	−15.9	2	18	—	**2**	**54**	11	15
1a_u_	−14.7	2	37	—	—	**48**	5	10
1b_u_	−14.5	2	**72**	—	—	5	21	2
1b_g_	−14.4	2	27	—	—	**50**	4	19
2a_g_	−14.3	2	**94**	4	**2**	—	—	—
3a_g_	−14.0	2	**47**	—	—	8	40	5
2a_u_	−14.0	2	**100**	—	—	—	—	—
2b_g_	−13.9	2	**53**	—	—	26	9	12
3a_u_	−13.6	2	30	—	—	32	—	**38**
3b_g_	−13.6	2	**100**	—	—	—	—	—
2b_u_	−13.6	2	**98**	2	—	—	—	—
4a_g_	−13.4	2	**77**	—	—	3	19	1
3b_u_	−13.3	2	**43**	—	—	18	37	2
5a_g_	−13.0	2	32	—	—	**44**	18	6
5a_u_	−12.9	2	31	—	—	**36**	26	7
4b_g_	−12.9	2	20			**61**	17	2
6a_g_	−12.7	2	26	14	—	**45**	6	9
4b_u_	−12.0	2	**65**	10	2	14	9	—
5b_g_	−11.4	2	3	—	—	**50**	10	37
6a_u_	−5.4	0	—	—	5	25	12	**58**

The G_2_Au_2_^2+^ cluster ion has 10 Au 5d AOs, which span a_g_ (1a_g_, 2a_g_, 3a_g_, and 4a_g_ MOs) + a_u_ (low 1a_u_, 2a_u_, and 3a_u_ MOs) + b_g_ (1b_g_ and the highest occupied 2b_g_ MOs) + b_u_ (1b_u_ and 2b_u_ MOs) representations in the C_2h_ symmetry (Figure S3 and Table 6). Suppressing the Au(5d) AO participation, one note that the lowest unoccupied 4a_u_ MO at −5.7 eV is the combinations of three sets of C(2p), N(2p), O(2p) AOs, and one set of virtual Au(6p) AOs. Mixing of 5d AOs of two gold atoms yields the 2a_u_ (−13.4 eV) σ bonding orbitals in the middle of the valence band. Due to the relativistic effects, the strong mixing of Au 5d and 6 s AOs in particular the 2a_g_ (−14.0 eV), 4a_g_ (−13.0 eV), and 2b_u_ (−11.6 eV) MOs leads to the significant Au 5d-6s hybridization. It is clear that Au 5d AOs play some role in the highest occupied 2b_g_ (−11.5 eV) MO as the Au 5d AOs and π_g_ bonding orbital of C(2p), N(2p), and O(2p) AOs counteract each other. From these analyses, the aurophilic interaction occurs mainly through the mutual stabilization of three sets of 5a_g_, 6a_g_, and 3b_u_ δ orbitals, two sets of 3a_u_ and 3a_g_ σ orbitals, and also a little stabilized by threee sets of 4b_u_, 4a_u_ and 2b_u_ MOs, and two sets of degenerate 3b_g_ and 4b_g_ MOs. It is worth mentioning that, the bonding interaction in Au_2_G_2_^2+^ cluster ion involves the valence electron configuration 5d^9^6 s^1^6p° rather than the nd^10^(n + 1)s°(n + 1)p° configuration at Cu or Ag in Au’s homologues. The result seems reasonable because the relativistic effects in the valence shells of gold atom stabilize the s-atomic orbitals (AOs) and destabilize the d-AOs, leading to the drastically reduced 5d-6s gap of Au, and then resulting in Au’s particular electron configuration in the metal-mediated base pairs complexes.

**Table 6:** Characters, Orbital Energies (in eV), and AO Contributions in % (Largest is Bold) of the Au 5d-derived MOs and Lowest Unoccupied MO of the Lowest-lying Isomer of G_2_Au_2_^2+^ Cluster ion.

MO	ε	occ	Au(d)	Au(s)	Au(p)	N(p)	O(p)	C(p)	H(s)
1b_u_	−16.9	2	10	—	**2**	**57**	8	16	7
1a_g_	−16.6	2	11	—	2	**50**	13	21	3
1a_u_	−15.0	2	34	—	—	**50**	5	11	—
1b_g_	−14.7	2	13	—	—	**61**	6	20	—
2b_u_	−14.6	2	**50**	—	—	8	39	3	—
2a_g_	−14.4	2	33	—	—	7	**60**	—	—
3a_g_	−14.0	2	**80**	20	—	—	—	—	—
2b_g_	−14.0	2	36	—	—	**41**	14	9	—
2a_u_	−13.8	2	11	—	—	43	2	**44**	—
3a_u_	−13.4	2	**100**	—	—	—	—	—	—
4a_g_	−13.1	2	30	—	—	**57**	7	6	—
5a_g_	−13.0	2	**81**	19	—	—	—	—	—
3b_u_	−12.8	2	**94**	6	—	—	—	—	—
3b_g_	−12.8	2	**64**	—	—	29	4	3	—
4b_g_	−12.8	2	**73**	—	—	21	5	1	—
6a_g_	−12.7	2	**57**	25		10	6	2	—
4a_u_	−12.6	2	**59**	—	—	18	16	7	—
4b_u_	−11.6	2	**57**	26	3	7	7	—	—
5b_g_	−11.5	2	10	—	—	**45**	10	35	—
5a_u_	−5.7	0	—	—	3	26	13	**58**	—

For G_2_Cu_2_^2+^ cluster dication, the HOMO (Cu d) → LUMO (G_2_ π) excitation can be classified as metal-metal-to-ligand charge transfer (MMLCT), while for G_2_Ag_2_^2+^ and G_2_Au_2_^2+^ cluster dications, the HOMO → LUMO excitations are able to be classified as ligand-to-ligand (LL) charge transfer. These charge transfer processes are different from those in mononuclear metal-mediated base pairs^28,31^, modified significantly by the presence of the Cu–Cu, Ag-Ag, and Au-Au interactions. It may provide valuable information for their absorption or photodissociation experiments.

The lowest-energy isomer 2A of G_2_Ag_2_^2+^ dication has larger HOMO−LUMO gap of 5.98 eV at the DFT level, as compared to Ag’s heavier (HOMO-LUMO gap nearly 5.80 eV) and lighter homologue (HOMO-LUMO gap nearly 5.58 eV). However, the bond dissociation energies (BDEs) of G_2_M_2_^2+^ cluster ions calculated based on the differences between the total energy of the parent ion (G_2_M_2_^2+^) and the total energies of all the fragments (G and M^+^) at the DFF/B3LYP level (G_2_M_2_^2+^ → 2 G + 2 M^+^) are 8.52, 5.66, 8.50 eV, for Cu, Ag, and Au, respectively. This indicates that the sum of N-M, M-O, and metallophilic interactions in G_2_Ag_2_^2+^ cluster ion is much weaker than those in Ag’s heavier and lighter homologues.

**Figure 2:**
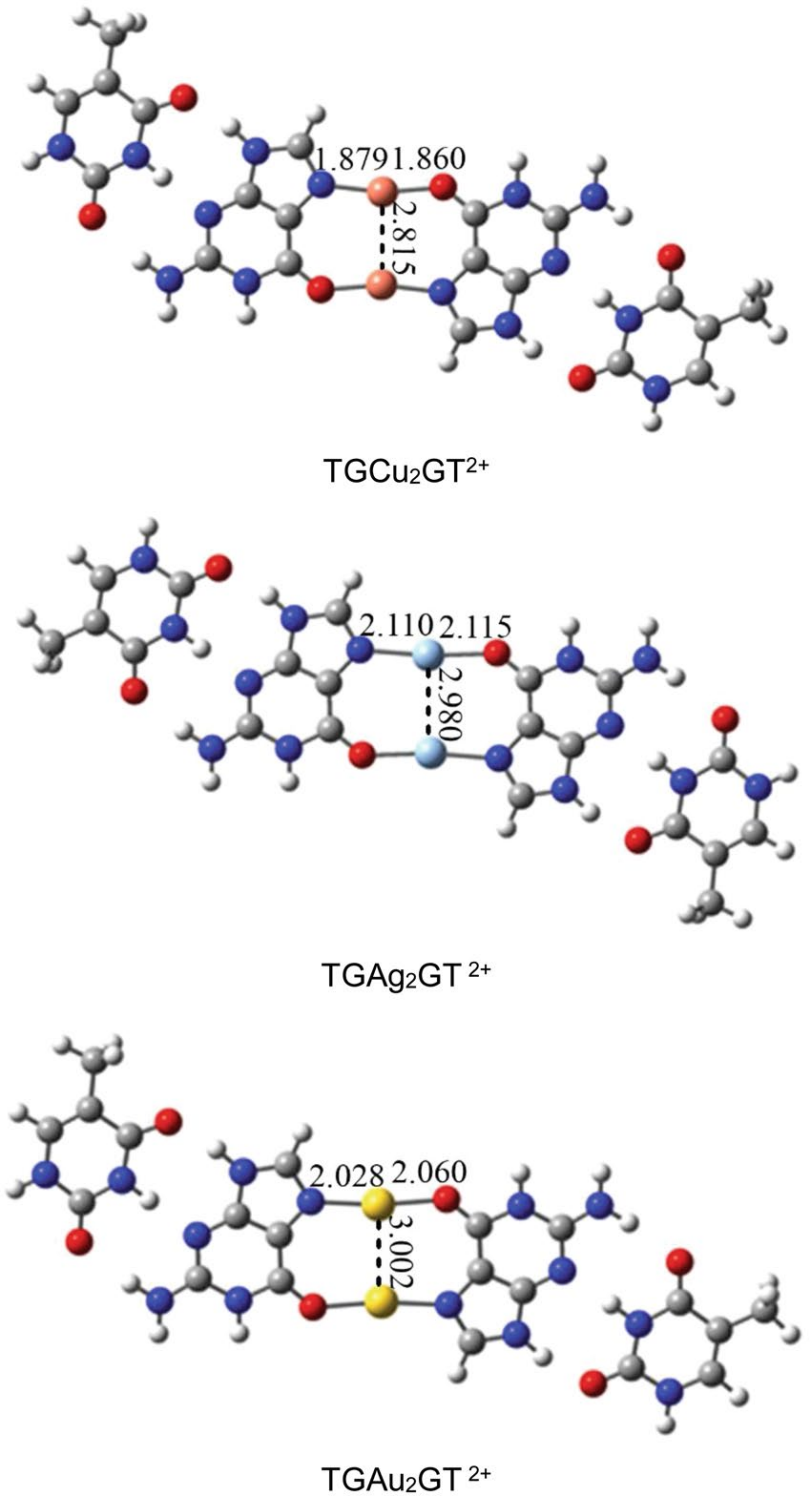
Structures and M-N, M-O and M-M bond lengths of TGM_2_GT^2+^ (M = Cu, Ag and Au) cluster ions.

Because dinuclear metal-mediated base pairs can be introduced as DNA sequences, it is interesting to compare the structural parameters of isolated G_2_M_2_^2+^ cluster ions with those of TGM_2_GT^2+^ (M = Cu, Ag and Au) cluster ions. Structures and M-N, M-O and M-M bond lengths of optimized TGM_2_GT^2+^ (M = Cu, Ag and Au) cluster ions have been shown in Fig. 2. The Cu-N and Cu-Cu bond lengths of TGCu_2_GT^2+^ ions have been slightly shortened relative to those of G_2_Cu_2_^2+^ cluster ions, while the Ag-N bond lengths of TGAg_2_GT^2+^ ion are slightly shorter than those of G_2_Ag_2_^2+^ ions. TGAu_2_GT^2+^ ion has slightly longer Au-Au bond length when compared with G_2_Au_2_^2+^ ions. Lengthened sequences containing G_2_Cu_2_^2+^ and G_2_Au_2_^2+^ cluster ions have slightly weaker metallophilic interactions relative to the isolated G_2_M_2_^2+^ cluster ions. However, lengthened sequences containing G_2_Ag_2_^2+^ cluster ions have almost the same argentophilic interactions relative to the isolated G_2_M_2_^2+^ cluster ions.

## Conclusions

The geometric and electronic structures of G_2_M_2_^2+^ (G = Guanine, M = Cu, Ag or Au) cluster ions have been investigated with DFT approaches. The lowest-lying isomers of the presently investigated species have C_2h_ symmetries with an approximately antiparallel alignment of N-M∙∙∙O groups being formed in the planar structures. Their HOMO-LUMO gaps significantly above 5.50 eV at the DFT level, indicating that these cluster dications are highly stable. The Ag-Ag, and Au-Au distances in these isomers are very similar, slightly longer than the Cu-Cu distance. Cu-Cu, Ag-Ag and Au-Au distances are shorter than the sum of van der Waals radii of corresponding two homo coinage-metal atoms, showing that metal-metal bonding can be characterized as weak but significant metallophilic interactions. From above-mentioned orbital analyses, s and d orbitals of metal atoms and p orbitals of C, N and O atoms all significantly participate in the orbital interactions. The compositions and bonding of the HOMO of G_2_Cu_2_^2+^ dication are completely different from those of the highest occupied MOs of Cu’s heavier homologues. The bonding interactions in Au_2_G_2_^2+^ dication involve the valence electron configuration 5d^9^6s^1^6p° rather than the nd^10^(n + 1)s^0^(n + 1)p^0^ configuration at Cu or Ag in Au’s homologues, although it may not be attached as an absolute physical meaning. The second lowest-energy isomers stabilized by an approximately parallel alignment of one set of O-M-O group and one set of N-M-N group are found to be close to the lowest-energy isomers in energy. The barrier between the two isomers of G_2_M_2_^2+^ cluster ions is significantly large, also showing that these lowest-energy isomers are very stable.

## Theoretical methods

To investigate the geometric structures and energetics of G_2_M_2_^2+^ (G = guanine, M = Cu, Ag or Au) cluster dications, DFT calculations were performed with Amsterdam Density Functional program (ADF 2013.01)^45–47^. The geometries were fully optimized using the generalized gradient approximation (GGA)^48^ of Purdue-Becke-Ernzerhof (PBE), converging to an energy gradient <10^−5^ Hartree∙nt Å^−1^ at an Kohn-Sham SCF criterion <10^−8^ a.u. The uncontracted Slater basis sets with the quality of triple-plus two polarization functions (TZ2P) were used, with the frozen core approximation applied to the [1s^2^-2p^6^] core for Cu, the [1s^2^-3d^10^] core for Ag, the [1s^2^-4f^14^] core for Au, [1s^1^] core for H and the [1s^2^] cores for C, N and O. The scalar relativistic was taken into account by the Zero Order Regular Approximation (ZORA). Optimized minimum structures were verified with nonexistence of imaginary frequencies in the analyses of vibrational frequencies. For more reliable energies, the single-point energy was calculated using the CAMY-B3LYP/TZ2P^49^ density functional method at the PBE/TZ2P geometries. The corrections of zero-point energy (ZPE) were also included in calculating the relative energies. To determine the atomic charges of Cu, Ag, Au, relevant N, and O atoms in these clusters, the population partitioning schemes of Hirshfeld^50,51^, Voronoi^52^, and multipole derived charges (MDC-q)^53^ were calculated.

The conductor-like screening model (COSMO) was also used to approximately explore the influence of a aqueous solvent environment on the geometries and energetics of the G_2_M_2_^2+^ cluster cations^54^. The following atomic COSMO-default radii from the ADF code were used: H 1.350, C 1.700, N 1.608, O 1.517, Cu 1.883, Ag 2. 025, and Au 2.025^55^. The parameters for water solvent were used in the COSMO calculations as water was the most common solvent used in the experimental studies for comparison.

The structures of G_2_M_2_^2+^ ions and transition states between two nearly degenerated isomers of G_2_M_2_^2+^ cluster dications were optimized using the GAUSSIAN 09 program^56^. The Becke 3-parameter-Lee-Yang-Parr (B3LYP)^57,58^ density functional method with the 6–31 + + G(d,p) basis set was chosen for nucleobases. The ECP10MDF, ECP28MDF, ECP60MDF relativistic effective core potential (RECP) developed by the Stuttgart-Cologne groups were chosen for Cu 1s-2p (3spd4s), Ag 1s-3d (4spd5s), and Au 1s-4f (5spd6s), respectively^59^. Gaussian type one-electron basis sets of MDF_VDZ were used for Cu, Ag and Au^59,60^.

## Electronic supplementary material

Supporting Information
