# Supplementary material for: Dinuclear Metal-Mediated Homo Base Pairs with Metallophilic Interactions: Theoretical Studies of G2M22+ (M = Cu, Ag, and Au) Ions
Source: Sci Rep. 2017 Nov 2;7:14896. doi: 10.1038/s41598-017-14259-2 (PMC5668421; doi:10.1038/s41598-017-14259-2)
Supplement: Supplementary file 1 — Supporting Information [file 41598_2017_14259_MOESM1_ESM.pdf]

Supporting Information

Dinuclear Metal-Mediated Homo Base Pairs with Metallophilic Interactions: Theoretical Studies of $G_2M_2^{2+}$ (M = Cu, Ag, and Au) Ions

Guo-Jin Cao*[,a]

[a]*Institute of Molecular Science, Shanxi University, Taiyuan 030006, China*. E-mail: caoguojin@sxu.edu.cn

Table S1 The optimized structures, and relative energies of low-energy isomers of $G_2Cu_2^{2+}$ cluster ion at the DFT/CAM-B3LYP//DFT/PBE level*.

ΔE	Symmetry	The optimized structures
0.00	C_{2h}	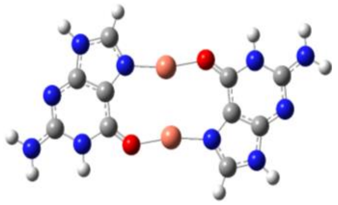
0.07	C_{2V}	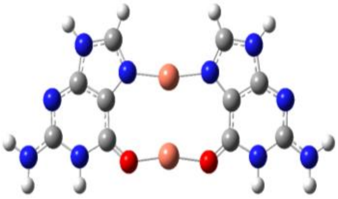
1.32	C_s	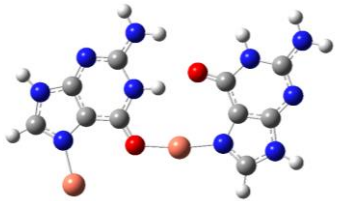
1.64	C_1	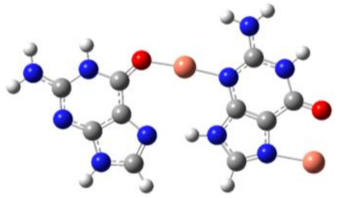
1.88	C_1	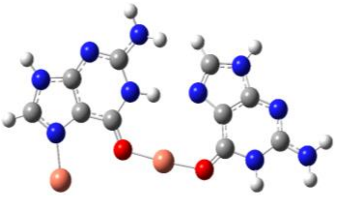
2.22	C_1	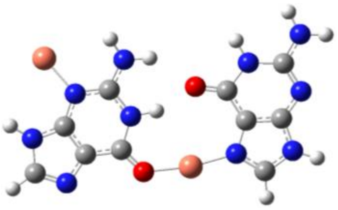

2.57	C_1	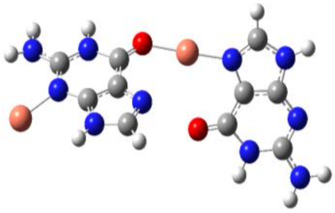
2.70	C_1	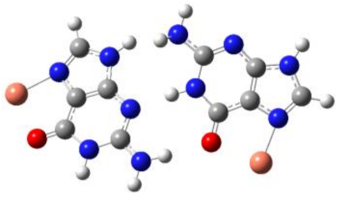
2.74	C_1	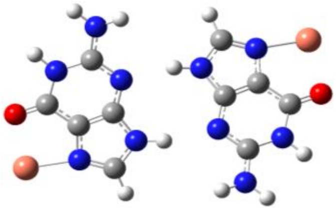
2.74	C_1	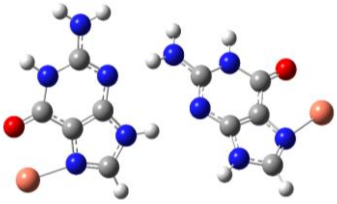
2.89	C_1	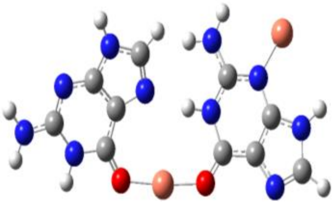
3.31	C_1	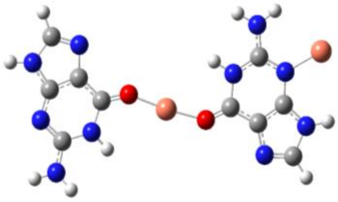
3.41	C_1	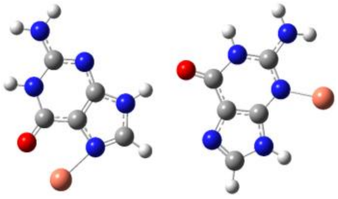
3.41	C_1	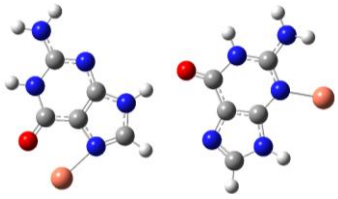

3.48	C_1	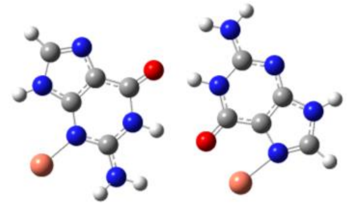
3.48	C_1	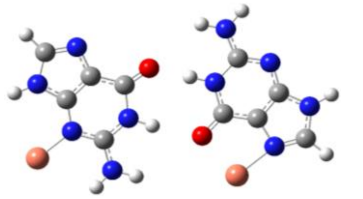
4.16	C_1	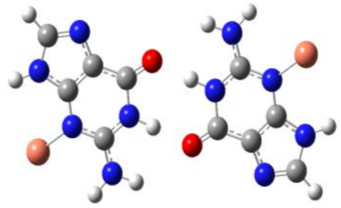
4.98	C_1	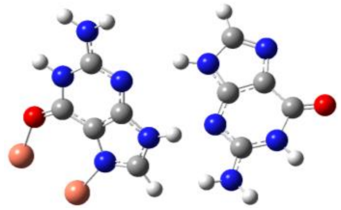
5.00	C_1	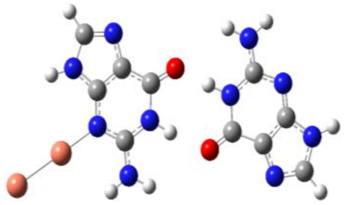
5.17	C_1	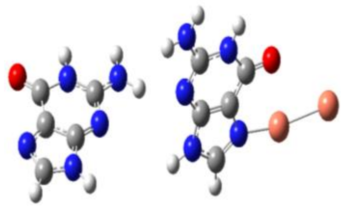

*The energies are in eV.

Table S2 The optimized structures, and relative energies of low-energy isomers of $G_2Ag_2^{2+}$ cluster ion at the DFT/CAM-B3LYP//DFT/PBE level *.

ΔE	Symmetry	The optimized structures
0.00	C_{2h}	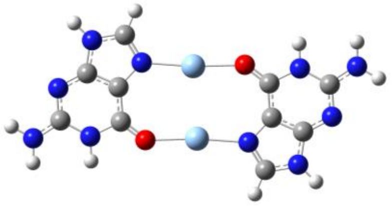

0.05	C_{2V}	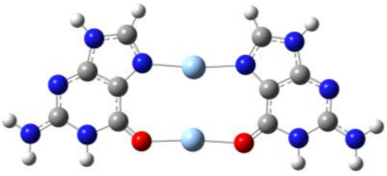
0.66	C_s	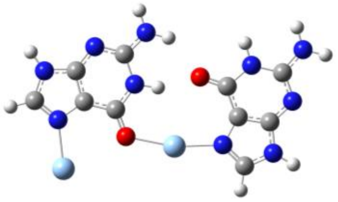
1.02	C_1	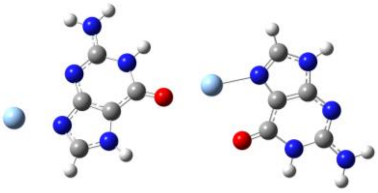
1.2	C_1	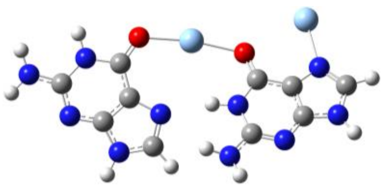
1.37	C_2	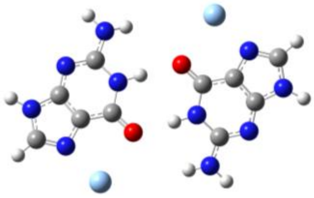
1.43	C_1	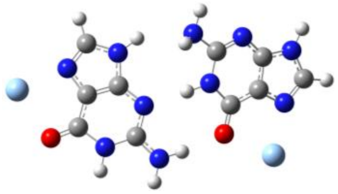
1.45	C_2	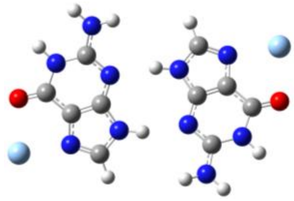
1.46	C_1	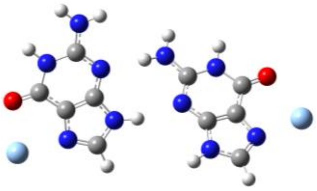
1.76	C_1	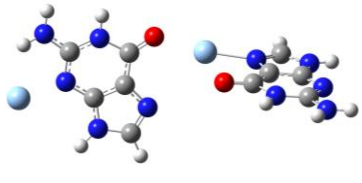

2.18	C_1	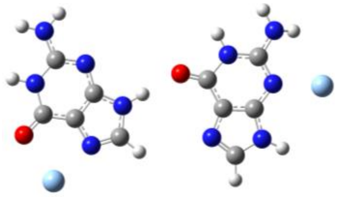
2.23	C_1	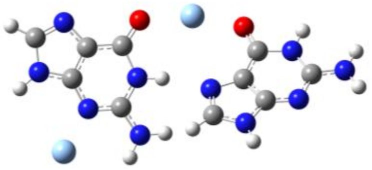
2.25	C_1	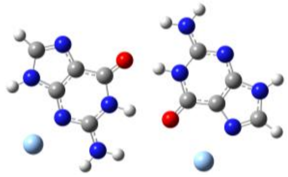
2.25	C_1	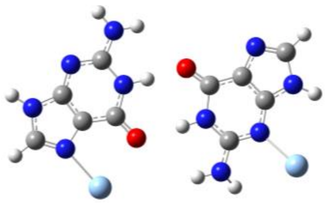
2.87	C_1	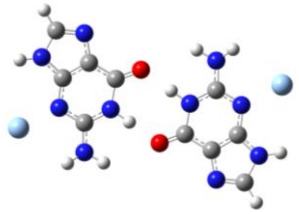
3.76	C_1	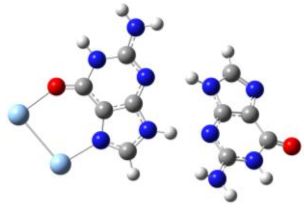
3.76	C_1	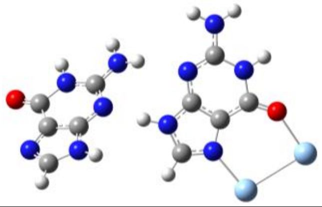
4.17	C_1	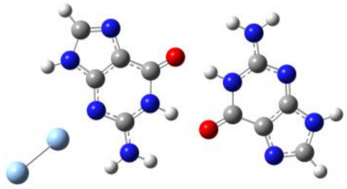

*The energies are in eV.

Table S3 The optimized structures, and relative energies of low-energy isomers of $G_2Au_2^{2+}$ cluster ion at the DFT/CAM-B3LYP//DFT/PBE level*.

ΔE	Symmetry	The optimized structures

0	C_{2h}	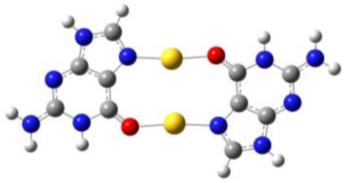
0.09	C_2	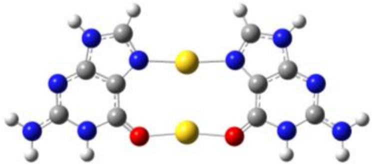
1.26	C_s	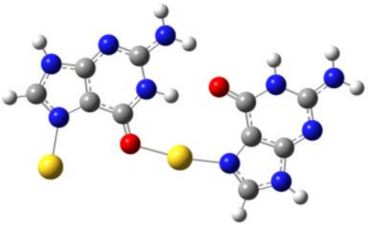
1.78	C_s	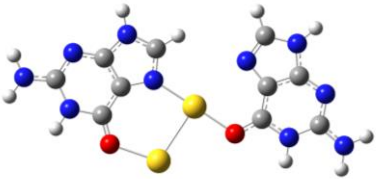
1.90	C_1	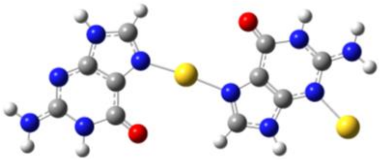
2.08	C_1	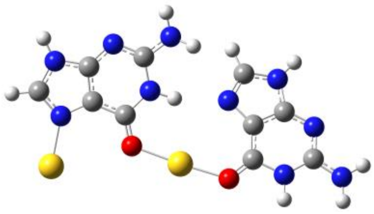
2.09	C_s	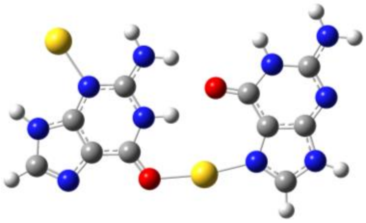
2.45	C_1	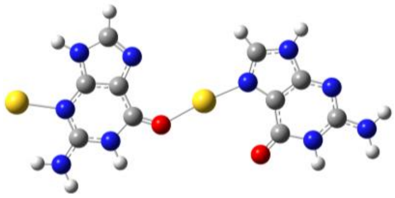

2.53	C_{2h}	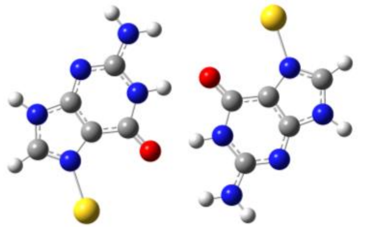
2.68	C_1	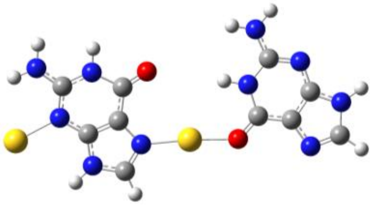
2.90	C_1	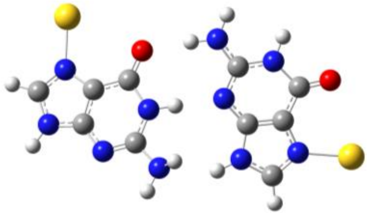
3.02	C_1	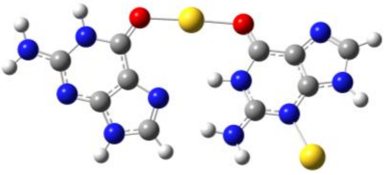
3.03	C_1	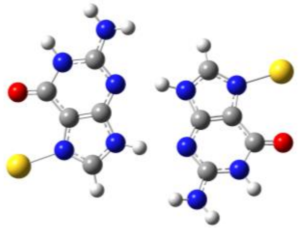
3.05	C_1	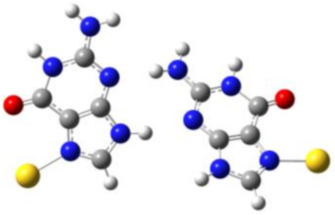
3.26	C_1	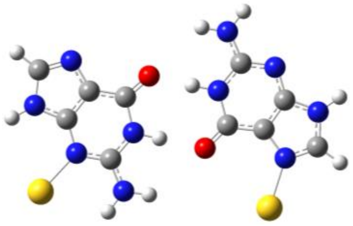
3.26	C_1	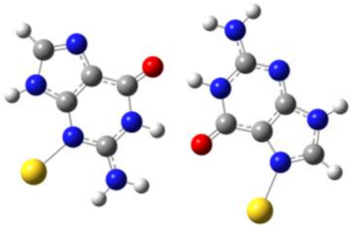

3.29	C_s	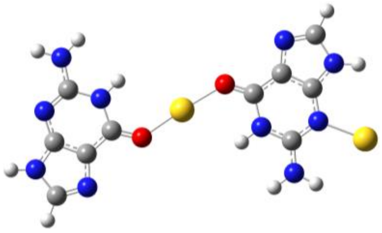
3.43	C_s	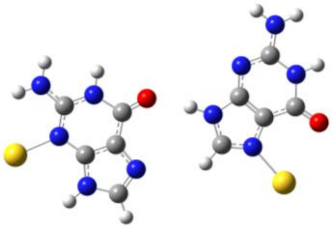
3.78	C_1	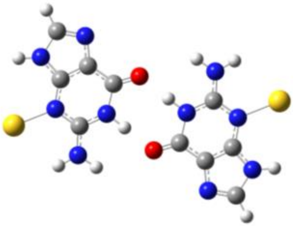
4.02	C_1	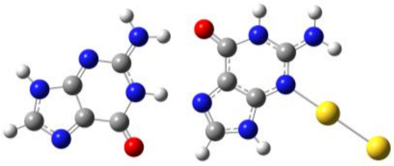
4.19	C_1	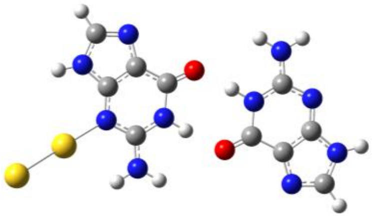

*The energies are in eV.

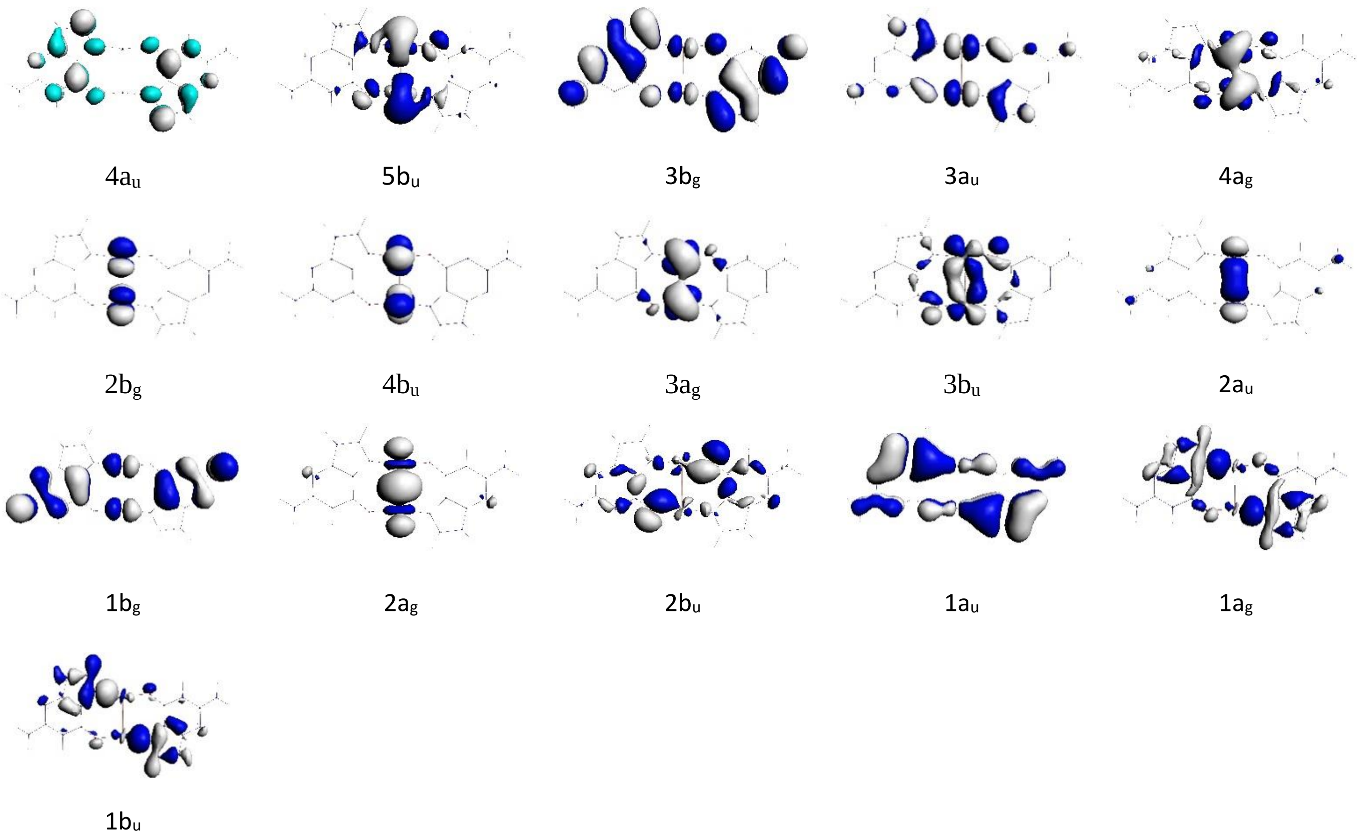

Fig. S1 Plot of lowest-lying unoccupied $4a_u$ MO and occupied molecular orbitals of $G_2Cu_2^{2+}$ cluster ion (isovalve=0.03).

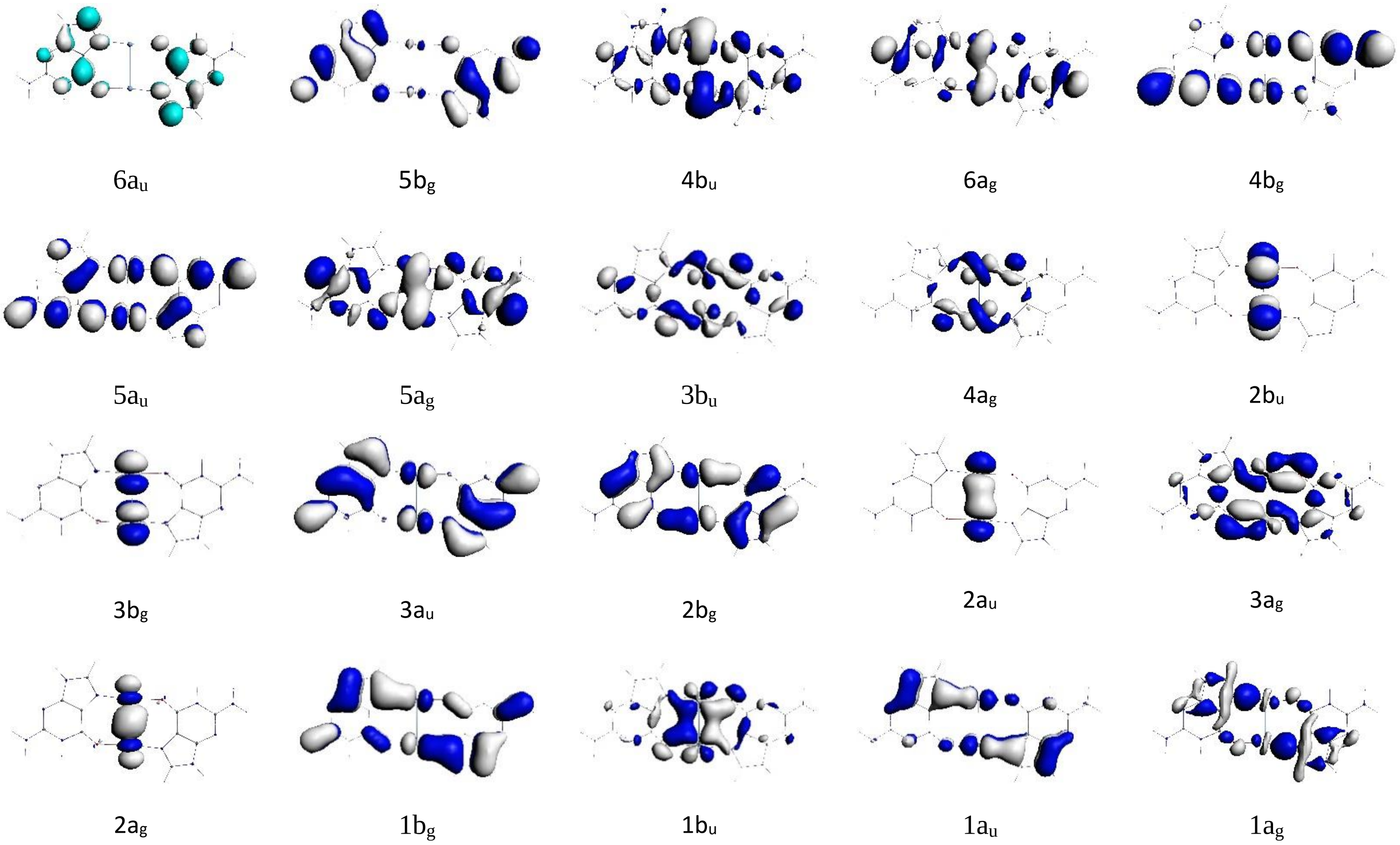

Fig. S2 Plot of lowest-lying unoccupied $6a_u$ MO and occupied molecular orbitals of $G_2Ag_2^{2+}$ cluster ion (isovalve=0.03).

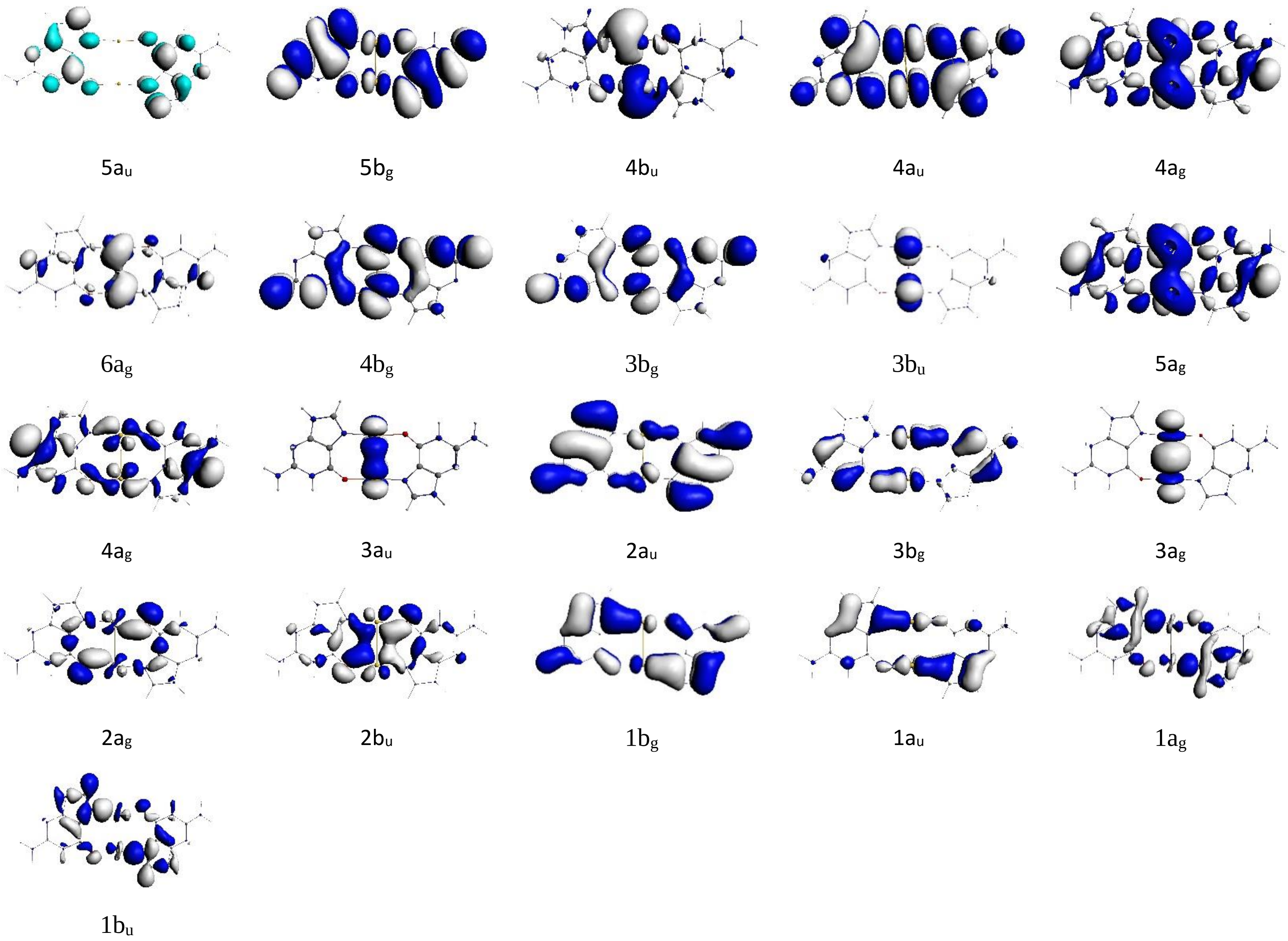

Fig. S3 Plot of lowest-lying unoccupied $5a_u$ MO and occupied molecular orbitals of $G_2Au_2^{2+}$ cluster ion (isovalve=0.03).
